# Lived experiences in help-seeking from the perspective of a mother with a dual diagnosis

**DOI:** 10.3402/qhw.v8i0.20316

**Published:** 2013-08-20

**Authors:** Minna A. Sorsa, Päivi Åstedt-Kurki

**Affiliations:** 1 School of Health Sciences, Nursing Science, University of Tampere and Finnish Doctoral Programme in Nursing Science, Tampere, Finland; 2 School of Health Sciences, Nursing Science, University of Tampere and Science Centre, Pirkanmaa Hospital District, Tampere, Finland

**Keywords:** Help-seeking, dual diagnosis, Giorgi, mental health, mental health services, mothers, phenomenology, substance abuse

## Abstract

Mothers with a co-occurring mental illness and substance abuse (dual diagnosis) use numerous different services. Help-seeking and engagement are complex processes which have not yet been sufficiently conceptualized. A descriptive phenomenological approach was used to explore these experiences from different service contexts and to describe the decisions in and structure of help-seeking over a 13-year period. Four in-depth interviews were conducted and data were analysed with a descriptive phenomenological method developed by Giorgi. The essential meaning structure is an inner conflict within the client, including a realization that change starts from within. The essential meaning structure combines the other meaning structures: disbelief of receiving help and admitting the need for help, keeping up the perfect façade and the risk of total collapse, being given and making own choices regarding care and being forced to use services and inner emptiness and search for contentment in life. It is possible that clients in the help-seeking process do not always recognize they have a need for care. If the client experiences inner powerlessness as emptiness and resistance to being helped, it is probably not possible to create relationships with care providers. Clients may have several ambiguous inner processes which prevent them from accepting the need for care. Theoretically and empirically a long-term approach is crucial, since the inner transformative processes take time. The services can contribute new experiences to the personal level of understanding and decision-making, if they consider the experiential level of their clients.

Persons with co-occurring mental health and substance abuse disorders are called dually diagnosed in the medical literature. They use numerous services and their care is considered problematic (Drake et al., 2001). Many people seem to have difficulties in linking and engagement with the services (Drake et al., 2001; Naegle, 1997; O’Brien, Fahmy, & Singh, 2009). There seems to be a contradiction in the fact that some people with mental health problems may not report any need for care, whereas others experience mental health problems and perceive a need for care (Beljouw et al., 2010; Rogler & Cortes, 1993).

Alonso et al. (2007) estimated that 3.1% of the adult population in Europe had an unmet need for mental health care. According to Sturm and Sherbourne (2001), 10.9% of the US population perceived a need for mental health or substance abuse care in a 12-month period. More than 25% of those with an unmet mental health need and more than 33% of respondents with unmet substance abuse needs either received no care or encountered difficulties in obtaining the care they perceived necessary. Beljouw et al. (2010) observed that 43% of people with anxiety or depression did not receive treatment and that the most important reason for not seeking treatment was a preference to manage the problems themselves. Avoidance of health care at any stage may result in individuals not receiving care and help (Clement et al., 2012).

No precise information is available on how many drug users live with children in Europe. About 1 in 10 clients entering treatment for drug use problems in 2010 lived with children (EMCCDA, 2012).

Little is known about why individual clients do not seek treatment (Beljouw et al., 2010; Clement et al., 2012; Rogler & Cortes, 1993). The actual reasons of dually diagnosed women for their decisions considering using services or not seeking help are not well known (Drapalski, Bennett, & Bellack, 2011). One reason for not seeking help may be another drug user in the family (Walton-Moss & Becker, 2000). The specific characteristics of women with a dual diagnosis include fear, background of abuse, experience of violence, and ambivalent emotions such as guilt and anxiety (Nehls & Sallman, 2005; Rosenbaum, 1979). Women with dual diagnosis have complex experiences. The literature has identified a controversy since women bear the primary responsibility for child-rearing, also in families with substance abuse (Grella, Polinsky, Hser, & Perry, 1999). Women may avoid entering care for as long as possible fearing that their children may be taken into care, and finally the times of admission are almost chaotic (Alexander, 1996; Howell, Heiser, & Harrington, 1998; Klee, 1998; Nehls & Sallman, 2005).

Dual diagnosis clients usually want to deny or minimize the extent of their substance abuse (Drake et al., 2001; NAIARC, 2005). It is known that substance abuse is a risk for dropping out of treatment (O’Brien et al., 2009) and that women with substance abuse disorders are likely to seek treatment in non-specialized settings (Greenfield et al., 2007).

A phenomenological perspective can reveal more about the decisions of individuals. Few studies have investigated in detail and in a long-term perspective why individual clients participate or disengage (after several years of engagement) (O’Brien et al., 2009). Wang, Berglund, Olfson, and Kessler (2004) noted that little is known about the patterns of initial treatment contacts, since help-seeking research has focused on recent service use rather than on the gap between initial help-seeking and treatment. As the clients become entangled in substance abuse over several years, the recovery also takes years (Drake et al., 2001; NAIARC, 2005). This means that a substantial amount of time may be required to engage and motivate dual diagnosis clients to use services (Drake, Mueser, Clark, & Wallach, 1996).

Even if a professional supports participation in treatment programmes, the clients themselves may consider participation irrelevant (Drake et al., 1996; Naegle, 1997; O’Brien et al., 2009; Rogler & Cortes, 1993). Also, service providers may miss clients due to ineffective working methods or inadequate assessment. If the clients were identified at an earlier stage, their time of recovery might also be shortened. Giving up drug abuse requires multiple episodes of care over several years (Dennis, Scott, Funk, & Foss, 2005).

O’Brien et al. (2009) claim that engagement in the services is a complex phenomenon encompassing factors including acceptance of the need for help, the formation of a therapeutic alliance, goal-setting with professionals and satisfaction with the help received. It is a complex phenomenon, which has not yet been sufficiently conceptualized. Barriers refer to the reasons why individuals do not utilize specialized services (Greenfield et al., 2007). If key barriers to care are identified, potential interventions to increase care seeking and service use could be developed, and the reduction of untreated illness would follow (Clement et al., 2012).

One example of an obstacle to care or seeking help is due to the fact that the dual diagnosis clients and their families rarely have good information about services (Drake et al., 2001; Clement et al., 2012). Villena and Chesla (2010) identified barriers on an interpersonal level, in navigating the health care system and in issues related to housing. The reasons for avoiding or delaying seeking help are numerous, but the current knowledge about barriers is not comprehensive (Clement et al., 2012).

The qualitative aspect can reveal an in-depth understanding of the use of services from the client’s perspective. In contrast to the early dual diagnosis literature (Lehmann, 1989/2000), more is known today about what is helpful from the client’s perspective. Specialized services and programmes have been developed, and the experience of the client (or consumer) has become a part of the evaluation of integrated service approaches (Minkoff, 2006). Listening to different lived experiences is important to uncover meanings and ideas that otherwise might go unnoticed, since there are many paths of entry (Nehls & Sallmann, 2005).

This is a study with an interest in the decisions and lived experiences in the lives of mothers with a dual diagnosis. The goal of this article is to develop knowledge about the individual choices made and whether there are barriers restricting the use of services in the client’s experienced lifeworld, the decisions in help-seeking and in relation to the services used. This article aims to explore and describe the lived experiences, intentions and motives in help-seeking from the perspective of a mother with a dual diagnosis.

## Methods

Descriptive phenomenology as described by Giorgi (1992, 1997, 2012) was used. Description is a clarification of meaning. The emphasis is on examining phenomena as they manifest themselves to consciousness. The meanings can be described in their ambiguity and complexity (Giorgi, 1992) just as the data presents itself. The unique experience enables an in-depth description of the experiences, intentions, and choices that have an impact on help-seeking. It is assumed that subjectivity can reveal valuable information on services, and will be of general public interest.

### Inclusion in the study

Inclusion in the study occurred at three different levels of service delivery, using a purposeful sampling technique (Kvale, 1996; Speziale & Carpenter, 2002). Three settings focussing on the care of families with substance abuse problems and mental illness were chosen: a psychiatric hospital ward and two substance abuse rehabilitation settings. The goal was to reach dual diagnosis mothers with long experience of using the services. The staff helped in recruiting by finding women with long histories of drug abuse and severe mental health problems. In fact, several women were interviewed and later one of them was chosen for this study. The excluded interviews will be used in another study.

It is the researchers who need to decide when there are sufficient data for analysis. Also, the inclusion technique has an impact on the quality of data. The inclusion method needs to ensure that the participants are experts in the area of research (Englander, 2012).

**Figure 1:**
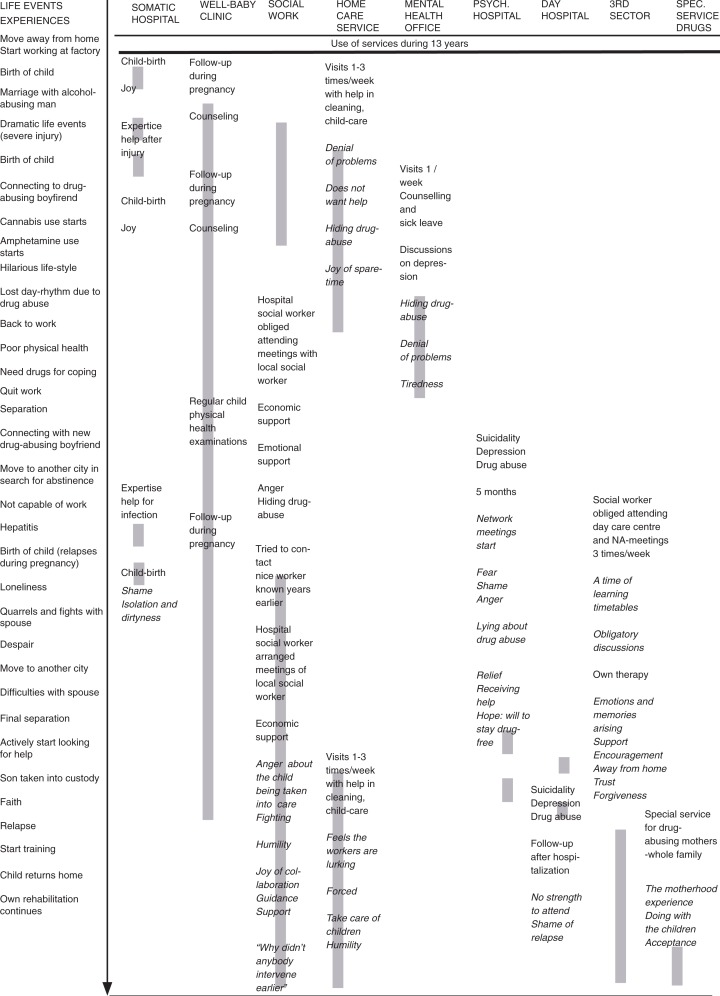
Life events (on the left), the use of services (grey lines), and the subjective experiences of using services (in cursive) during 13 years.

This mother’s experiences were chosen for this in-depth analysis because the data are rich, complex, with a revelatory content, contain extreme uniqueness and give the opportunity to use a deep and long-term perspective on help-seeking (Friberg & Öhlen, 2007; Hilliard, 1993). Giorgi (1997) stated that the resulting meaning structure can be based on one or several subjects. It depends on the research question whether to use several participants. However, Giorgi’s (2006) arguments for not choosing a single person are that the analysis may be stuck on a single subjective level, whereas a phenomenological description should be directed towards the phenomenon of interest. In our analysis, the subjective experiences of service use are shown in Figure 1. The meaning structure is captured in Figure 3. According to Kleiman (2004), Kvale (2007), and Englander (2012), the researcher adds participants until the needed data requirements are met. At the practical level, Englander (2012) says that frequently at least three participants need to be interviewed in order to reach a general meaning structure. We argue that data quality is not a choice about the number of participants, but depends on whether there is “sufficient” data, whether the interview and data analysis were performed systematically and whether the research aim can be reached. The goal of analysis in a complex context may not be to reach a general meaning structure, but to reach a structured understanding of a phenomenon (Giorgi, 2002). The inclusion of a single participant can be seen as a limitation of the study. Yet, the context is utterly complicated, which is an interesting aspect of inclusion strategy.

### Ethical considerations

This study was approved by ethics committee obtained from three different service providers to guarantee the anonymity of the participant (City of Tampere, Dno SOTE:/5407/403/2003; Tampere University Hospital, R03125H and an approval from the A-Clinic Foundation). Staff members gave the participant written and oral information on the study before being asked by the first author (M. S.) to give her written consent to participate. As the interviews contained emotional reminiscing in sensitive areas, they were conducted in specific settings, so that contacting a key worker would have been possible at the time of the interviews. The interviewer is a trained mental health nurse, so it was established that the research interview did not have therapeutic goals (see Kvale, 2007). The participant was given an opportunity to withdraw from the study at any stage. Participation did not have any impact on use of services. All data were handled by the first author only to ensure confidentiality. The article was read and approved by the subject herself, and she agreed to publication.

### Interviews

Four interviews were conducted in order to achieve a trustful interviewing atmosphere, and to enable a full description of the individual experiences. Kvale’s (1996, 2007) open-ended, thematic and discussion-like qualitative interview method was used. The aim was to allow the participant to talk freely, so the interviewer, who is a trained mental health nurse, did not make interpretations, but asked for clarifications. The unique life situation provides a context and the meanings are context-dependent (Englander, 2012). The interviewer wanted to have an answer to her research question, so she checked that all areas in the list of themes were covered (Kvale, 2007). The open-ended interviews were focused on themes and the participant was encouraged by questions and probes, such as, asking to tell more about a certain experience (Table I). Bracketing, setting aside previous ideas of the phenomenon of interest (Giorgi, 1997), was used already when planning the study, so the interviewer was focused on not taking anything for granted. During the interviews, the phenomenological level was not lifted up by the interviewer. The complexity of the subjective experiences could be revealed during the four tape-recorded interviews conducted at the end of 2003, approximately 1 week apart and over 1 month. Each interview lasted for 80–120 min. The interviews were partially transcribed verbatim prior to the next interview. On a practical level, the participant’s life events were discussed several times and data from different interviews were mutually reinforcing, making it possible to step into a more trustful discussion between the participant and the researcher.

**Table I:** Themes of the interviews.

Interview no.	Themes
1	Learning to know the participant and developing trust. The individual and her family, the social network, background and education, abuse of substances, mental health.
2	Family everyday life: success and times of trouble in relation to taking drugs and mental health.
3	Growth into motherhood and life in relation to drug abuse and suicidality. The many roles.
4	Help and support received and discussion on themes arising from the earlier interviews. Ending the collaboration.

### The participant

The participant of the study has a long history of drug abuse and mental health problems; she has used various services (Figure 1). Her life story is described through core life events and her use of different services.

She has three children, all of whom were living with her at the time of the interviews. Earlier, one of the children had been taken into care for a period of time. This mother took amphetamine and cannabis for several years without any professionals intervening in her lifestyle. During this time, she attended somatic hospitals, her children had follow-ups in well-baby clinics, she had contact with social workers and home-care workers actually visited her home. She sought help for different reasons. Her third child was born at a time when her drug abuse had increased. At times, her drug abuse was chaotic. She was depressed for several years and considered committing suicide prior to actually engaging with the services.

Changes in her life occurred over a long time period. There is an actual phase after which she committed to care through psychiatric hospitalization. She entered the services in a crisis situation with a total collapse of her lifestyle.

She used many rehabilitative services after two phases of psychiatric hospitalization. The phase of actual engagement and recovery included a process of thorough work on mental health issues, substance abuse, own personal growth, motherhood issues and relationships. At this stage, she received many parallel services and outreach services such as home-care services. The system obliged her to adhere to certain routines, for example, attending Narcotics Anonymous groups. At the time of the interview, she had actively used the help of different service providers and had been in a recovery phase for the past 4 years. She had started vocational training.

### Data

The interviews were transcribed verbatim; the data consist of 90-page text. The interviews cover the participant’s life history, but as the interest in this study was in the use of services, a limit was set to this content. The meaning units for analysis were strings of words of relevance to the study. A new meaning unit was created when the researcher noticed a transition in meaning (Giorgi, 1997). Examples of original meaning units are shown in Table II.

**Table II:** Examples of data.

**Example of original data extract:**
*It was then that I realized that drugs are taking me 10-0. So, so it was as for motherhood the worst time, so I could not any more. I could not so sort of hold together the coulisse, together, and I did not endure to use drugs, and I did not endure being without either, and then I sort of nestled and sort of. So I have always been very sociable and a lot dealing with other people and then I nestled sort of, in a sort of, so I was just alone at home and I wept and the children saw me crying and I felt really bad and I was quite irritated on myself. And I just went on destroying myself, so of course I also at the same time destroyed my children at the side, and then when you want to be a good mother, and I believe that everybody wants to be a good mother, so, so it was so contradictory situation inside myself. And so I wanted something else than I then acted, that sort of gnawed extensively and then started to grow, that I want to die and I want my children out of this awful world and I was as a failed mother*.
**Examples of thematized original meaning units:**
“*I want to be a good mother and I do not want to use drugs*”
“*So then I didn’t anymore have the strength to be a mother, I was so disappointed into what I wanted to be and then I could not manage to be like that*”
“*It was such a disappointment to me that when I was so, everybody wants to be a good mother and so so that, then so that I also had to use during, it was so that so that, hey what is it with me?*”
**Examples of researcher’s reflexion (notes) on thematized original meaning units:**
*A controversial awakening?*
*Contradictions and movements?—contradictory convalescence—motive is born?*
->*Researcher looked for other similar examples in data*
**Examples of meaning units and original data extracts (disbelief of receiving help and admitting the need for help):**
*I was irritated by people who felt sorry for me*
*I can manage, and that also comes from childhood, as I have been forced to cope*
*So I didn’t really accept help from anybody, and that kind of things*
*You need to manage, good girls don’t cry, you have to go forward in life*
*It changed into positivity; they really want to help me, why don’t I give them a chance?*
*I needn’t always manage by myself*
*I gave myself permission to invest in myself and my care*

There are 501 meaning units describing hospital care, 404 describing the use of other care provision and contexts, and 39 describing the children’s service use.

### Data analysis

**Figure 2:**
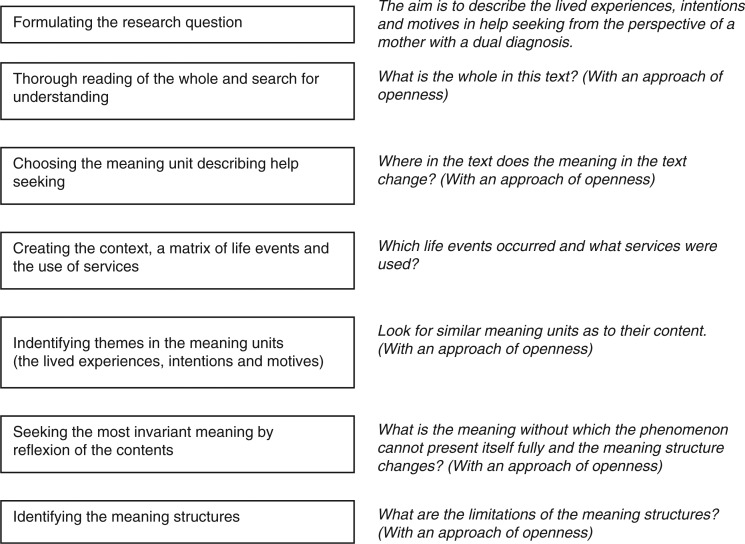
The analysis process and questions guiding the researcher.

Data were analysed by Giorgi’s (1997, 2012) descriptive phenomenological method. The first author used an attitude of attentive openness throughout the analysis (Kleiman, 2004). The process and questions guiding the analysis are described in Figure 2.

The research question was formulated to be suitable in a phenomenological study. The research question was guiding the steps of the data analysis. The transcribed text was read as a whole to get an overall sense of the data. Thereafter, specific meaning units in regard to the research questions and the experiential level were highlighted. If the meaning in the original text changed, a new meaning unit was marked. First, to grasp a sense of the complexity of the services as the context of help-seeking, the meaning units were classified according to the use of the service delivery system where contacts occurred. A matrix of life events and the use of services were created (Figure 1). This is what Giorgi (1997) refers to as the contextual factor.

Second, the meaning units were classified inductively with the research question as guiding the analysis process. Similar expressions in the mother’s own words were grouped together and thematized and later during the abstraction process categorized according to their content. The attitude towards data is the key in the access to subjectivity (Giorgi, 2012). It was necessary to look at the data from a different perspective than in an everyday life approach, and withholding pre-existing claims about the results (Giorgi, 1997). The meaning structures of the intentions were formed via freely reflecting the content of the meaning units (Kleiman, 2004). Thus, the phenomenological level was revealed. The phase of searching for most invariant meanings of each theme revealed the final results, as the meaning structures have an internal complexity. The question at this stage was: What is the meaning without which the phenomenon cannot fully present itself? It was necessary to go back to the original data; an inner constant and sensitive dialogue between actions, decisions and meanings.

### Trustworthiness of the study

Giorgi (2002) mentions validity as a consistency arising from an order of meanings, the contradictory question is that “something could always be other than the way it is.” In order to be consistent and to avoid interpretations within data, phenomenological bracketing was used by writing down earlier understandings and preconceptions of the phenomena prior to the analysis (Giorgi, 1992; Kleiman, 2004). In the interview phase, the subject–subject relation was altered into a subject–phenomena relation at the stage of the analysis (Englander, 2012). Giorgi’s (1997) requirements for a successful Husserlian descriptive study were followed: (1) only using description, (2) using the attitude of phenomenological reduction throughout the study, and (3) seeking the most invariant meanings for a meaning structure (Giorgi, 1997). The data collection and analysis are in line with the research methodology. The analysis process was systematic; the researcher’s attitude during data analysis and reduction was that of looking at reality as openly as possible without closing any doors in advance (Kleiman, 2004). The decision trail has been described, which is a key indicator of trustworthiness in a phenomenological study (Koch, 1994).

The researchers looked for themes emerging from the textual data (Koch, 1994), trying to reach genuine insights with no prejudgments (Giorgi, 2002). Trustworthiness is achieved by letting the experience present itself in its entirety full. The researchers tried to grasp meanings in line with the research questions as well as the scientific orientation (Giorgi, 1997). The end result is a descriptive report revealing the context on an emotional level and the life choices, the actions (Figure 1) as well as the inner intentional level (Figure 2). The mental health questions, the use of substances, and motherhood are the context of the unique experiences in help-seeking. The methodological choices can be defended because of the possibility for an in-depth description.

## Results

**Figure 3:**
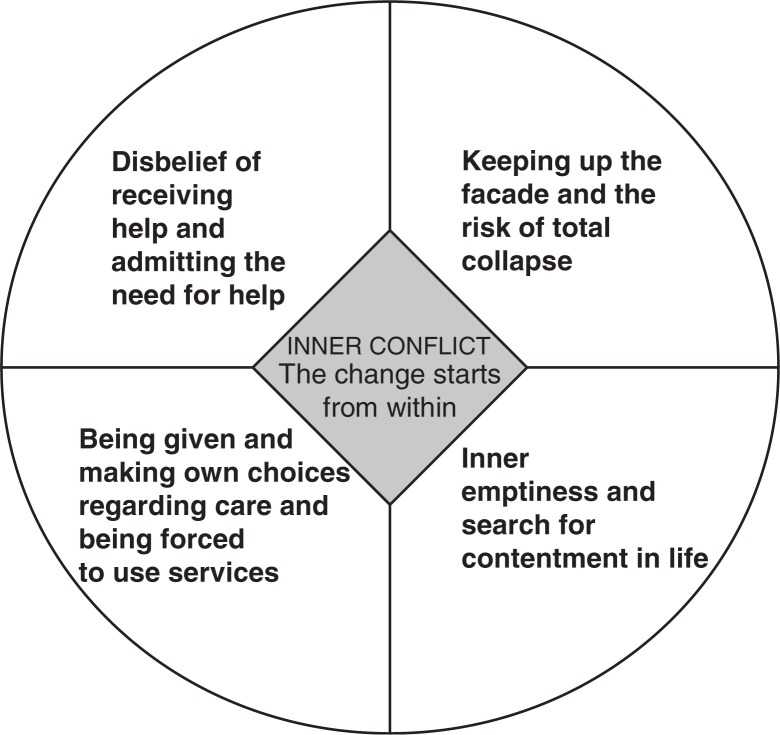
The lived experiences, intentions, and motives in help-seeking from the perspective of a mother with a dual diagnosis.

The results will be presented through the meaning structure of the intentions and motives in help-seeking (Figure 3).

The essential meaning structure is an inner conflict within the client, including a realization that change starts from within. The essential meaning structure combines the other meaning structures: disbelief of receiving help and admitting the need for help, keeping up the perfect façade and the risk of total collapse, being given and making own choices regarding care and being forced to use services and inner emptiness and search for contentment in life.

### Inner conflict—change starts from within

The core in help-seeking experiences seems to be a certain state of inner conflict, which is dwelling in all the themes and in the structure of the inner experiences (Figure 2). All meaning structures exist concurrently. There are inner levels of experiences that have an impact on help-seeking and the perceived barriers.

The inner changes seemed to create motion via extreme life situations of contradiction. For example, being eager to breast-feed the child for a long time, and yet relapsing when the child was 2 weeks old. This resulted in an inner conflictual time period when changes grew out of ambivalence. As another example, it took her several years to free herself out of quarrelsome family life that she did not anticipate.

All the meaning structures involve conflict and issues needed to be solved on an experiential level. Her insight was that all change starts from within. The inner processes were needed, so that committing to care became an option.

### Disbelief of receiving help and admitting the need for help

The participant felt annoyed at people who wanted to help her, or whom she regarded as feeling pity for her life situation. The experience of disbelief of receiving help is an inner choice, and creates a barrier to the use of services. She preferred to manage her problems herself.

The explanations were often rooted in her background, where she “had too much freedom” and was obliged to take care of herself very early in her life course. The experience of disbelief in receiving any help was rooted in a deep-seated mistrust of the workers in the social and health care services. She claimed that she was incapable of letting anybody come too close to her as a person.

Contradictory to the experience of disbelief of receiving help, she admitted the need for help. It started from a trustful relationship with a psychiatric nurse on a hospital ward. An attitude of humility in relation to admitting her drug abuse appeared.

She needed to start a period of her own care before she could focus on her children. Accessing the services was a manifestation of the need for help. A family member helped.

### Keeping up the perfect facade and the risk of total collapse

She perceived herself as a mother of action, who kept her home clean and tidy, and dress her children in nice clothing. It was as though she sought external perfection. Initially, drugs gave her the energy to live up to her high ideals of motherhood.

Actually this created a barrier, because starting to take drugs meant a choice of secrecy and keeping up a social façade. The truth would have meant a risk of an experiential collapse. Therefore, she wanted nobody to interfere with her lifestyle. She had started to use drugs briefly after the birth of her second child. The façade was kept up by concealment. At the start taking drugs was fun, as the sad feelings and difficult memories of her past were forgotten, and drugs gave her the energy to clean, work, and play.

However, she felt powerless. Drug taking had started in the evenings when the children fell asleep. As time went by and her toleration grew, she needed more drugs. She became more tired even physically, and lacked the energy for everyday cleaning. The life rhythm changed, and she was awake in the night and felt sleepy in the day. She was prescribed medication for depression.

### Being given and making own choices of care and be forced to use services

She and her family used different services (Figure 1) according to their own choice for 9 years. One reason for avoiding the services and keeping the secret was the fear of the children being taken into care. This fear became a reality at a psychiatric hospital when a social worker enforced the taking into care of one of her children. When her story became known to local social workers, she lost her freedom of choice and the services assumed a stronger role in counselling. The system enforced the use of specific services, and she was not allowed to choose them. She felt discouraged by the service system, but found out that adjusting to the rules was necessary so that she could regain custody of one of her children.

The freedom of choice in using services returned gradually during her rehabilitation process. She analysed her change and feelings of shame as a necessity to become humble, in order to be able to move forward in her recovery. Relationships developed with personal workers, and the recovery could proceed.

She realized that she had actually needed the strict regulation and external barriers during the process to find out about her internal barriers. Her own choices and freedom had created barriers to help-seeking, and controversially the experienced total collapse resulted positively in care.

### Inner emptiness and search for contentment in life

When she finally found herself to be free of substance abuse, an inner emptiness emerged. The actions she had taken turned out to be actions experienced as inner emptiness. Energy had been devoted to concealment and keeping up a façade.

The experienced inner emptiness manifested in actions: she had not felt inclined to reveal her life events to care providers. She searched for contentment in her life. The children were a motivating force, who experienced life along with their mother. She realized the importance of motherhood in a care setting where the focus of care was solely on herself, and not on her as a single mother with children. She required more, and came to the realization she had actually not been a good mother during her drug history and bouts of depression. It was an inner experience of failure to provide the children with security. Rehabilitation started as motherhood became of more interest and her resource.

When she was taking drugs, her life involved experiences of threat. After the choice to stay drug-free, the service system assisted her in not contacting drug-abusing friends. Slowly, a new hope grew in her via religious companions, and gradually she could feel gratitude towards past events in her own life. It was an inner journey towards becoming more aware.

Finally, therapy gave her a new life course. She learned feelings she had not known even existed. There were emotions that she had fought against. With support, she could see her difficult life events in a new light. One form of freedom was forgiveness. She thought that help from others was needed to go through this process. Finally, she experienced meaningful contentment in her life.

## Discussion

The results show that there are both inner possibilities for help-seeking and engagement, as well as inner barriers impeding help-seeking. This sensitive inner level with the possibilities to change and recovery will be discussed. First, we will discuss the sufficiency of the data with a single participant and four in-depth interviews with a retrospective approach.

### Method

Methodologically, setting the goal and aim of the study were a significant starting point of the study. This article aims to explore and describe the lived experiences, intentions, and motives in help-seeking from the perspective of a mother with a dual diagnosis. In order to grasp a sense of the full individual experience, it is relevant to look at any client’s life history in a long-term perspective (several years). Giorgi’s descriptive phenomenological method was followed. The method allowed for an in-depth approach and new insights.

The phenomenological reduction with bracketing and openness were the clues to finding the inner structures of the lived experiences, intentions, and motives in help-seeking. The phase of the search for the most invariant meanings revealed the complexity of the experiences of mothers with mental health and substance abuse problems in relation to help-seeking and engagement in care. The descriptive phenomenological approach allowed for a deep dive into the individual experiences.

We described the help-seeking process and services as a Life matrix (Figure 1), which is not a method described by Giorgi. As the help-seeking process is complex, we wanted to help the reader to grasp the context of the service use from the perspective of a mother with a dual diagnosis. Essentially, this figure shows the choices, actions and emotions that came up in the interviews. Yet, this level of analysis is not the phenomenological level of meanings. We did not analyse the individual in relation to the background context of fragmented services.

As the context was a complex service delivery system, we think it was sufficient to have one participant only, since the data were so rich. Giorgi’s (2006) critique for not choosing a single person’s data for analysis is based on his doubts about whether a phenomenological description will be possible, without focus on the subjectivity. In our analysis, the subjective experiences of service use are shown in Figure 1. The meaning structures also contain subjective examples (Figure 3).

According to Giorgi (2002), the researcher needs to understand the conditions under which valid knowledge can be obtained. Data quality is not a choice about the number of participants, but depends on whether there is “sufficient” data, how the process of inclusion to the study succeeded, whether the interview (in this study with open-ended probing questions) and data analysis were performed systematically, whether several interviews were conducted, and whether the research aim can be reached.

The goal of analysis in a complex context may not be to reach a general meaning structure (which would require several participants), but to reach a structured understanding of a phenomenon (Giorgi, 2002). Five meaning structures were identified, as well as an essential meaning structure (Figure 3). The structures are essences and relationships (Giorgi, 1997).

The inclusion of a single participant can be seen as a limitation of the study. Yet, it can also be an advantage in the research viewpoint that it allows: the life situation as well as the context is utterly complicated. The strength of the phenomenological method is tying separate levels into a whole. As this study focused on a complex phenomenon basing on a single case, more studies are required to learn more about dually diagnosed mothers’ and their families’ subjective experiences of help-seeking.

As to the decision trail used (Koch, 1994), we have presented the interview themes (Table I), examples of original data, examples of thematized original meaning units, examples of researchers reflexion on thematized original meaning units and examples of original data from a certain meaning structure (disbelief of receiving help and admitting the need for help) (Table II) and the analysis process (Figure 2).

### Service use experiences

The data contain very interesting details in regard to service use: the services are multiple, sequential, and do not connect with each other except in the last 4 years (Figure 1). The client’s own will and choice was to conceal her problems, and the service system lacked means to perceive the situation. The services can contribute new experiences to the personal level of understanding and decision-making.

According to Deegan (2003), the individual needs and goals are complex, and the visibility of the complexity should be secured also in research.

The argumentation of the participant included the classic fear of the children being taken into care (Howell et al., 1998; Klee, 1998; Nehls & Sallman, 2005), and thus concealing the drug abuse (Drake et al., 2001; NAIARC, 2005). These data suggest that the process is much more complicated, and more research is needed on the impact of the personal experiential level of dually diagnosed women seeking help (Drapalski et al., 2011). As much of the social support comes from informal social contacts (Trulsson & Hedin, 2004), also the non-specialized settings also need more research (Greenfield et al., 2007).

## Meaning structures

There are five meaning structures (Figure 3): inner conflict—change starts from within, disbelief of receiving help and admitting the need for help, keeping up the perfect façade and the risk of total collapse, being given and making own choices regarding care and being forced to use services, inner emptiness and search for contentment in life.

The data raise the question as to what engagement and early identification actually means in the care of the mothers with a dual diagnosis. The clients have the right to decide what they tell the service providers. This is an issue of personal autonomy, and the right to make choices.

### Inner conflict—change starts from within

The core in help-seeking experiences seems to be a certain state of inner conflict, which is dwelling in all the themes and in the structure of the inner experiences (Figure 2). All meaning structures exist concurrently. There are inner levels of experiences that have an impact on help-seeking and the perceived barriers. The inner changes seemed to create motion via extreme life situations of contradiction.

Her insight was that all change starts from within. The inner processes were needed, so that committing to care became an option. These data show that the implementations of the stages of treatment model (Mueser & Fox, 2002) might have been an option in addressing the inner conflicts. Mueser and Fox (2002) continue to say that recovery from dual disorders occurs via four stages, which are all categorized by unique motivational states. The engagement stage may take several years. As the client motivation changes in the different stages, this might be a tool for improving the accessibility of the services.

### Disbelief of receiving help and admitting the need for help

The data contained material suggesting that dual diagnosis clients have difficulty in linking with the services (Drake et al., 2001; Naegle, 1997; Villena & Chesla, 2010). A personal inner process was the main reason for concealment, not seeking help, and not engaging. Alexander (1996) has noted that women resist entering care as long as possible, which might lead to chaotic phases of entering treatment. Any moment when the client enters the services is valuable and requires the full attention of staff in the service system. Entry and relationship to care can create barriers (Villena & Chesla, 2010). There is a need to find out more about the decisions and explanations regarding service use (Beljouw et al., 2010; Clement et al., 2012; Greenfield et al., 2007).

### Keeping up the perfect facade and the risk of total collapse

The facades could be retained partly because the service delivery system did not screen and assess the family perspective, nor the abuse of substances. According to the literature, these areas are poorly developed among the services for the dually diagnosed (Drake et al., 2001; Minkoff, 2006; NAIARC, 2005). The services used in somatic hospitals and primary care would have been possible sources of proper assessment.

### Being given and making own choices of care and be forced to use services

The data included material on a search for perfection and a desire to hide inner vulnerable aspects. The literature claims that early detection is crucial. Here it was not possible, because the client made her own choice to maintain secrecy. She claimed that participation in services was irrelevant at such a time when she did not perceive that she had any problems. Linking and engagement with services can take a substantial amount of time, and professionals may view the client’s life situation differently from the client. NAIARC (2005) suggests a long-term approach in the care of women with dual diagnosis.

### Inner emptiness and search for contentment in life

An obstacle to care was an experience of inner emptiness, which did not support the search for treatment and solutions using the service delivery system. Drug abusers need to leave their former identity in the process of recovery (Trulsson & Hedin, 2004). The whole personal and social history plays a role in the perspective of long-term recovery (Deegan, 2003). As inner conflicts, there was a constant desire to achieve good motherhood vs. the client’s own emotions and chances of failure. This is line with Trulsson & Hedin (2004), who note that the evolvement of the female identity and growth of self-esteem are relevant. Motherhood was the essential empowering source. Some services only took the mother into consideration as an individual. Yet, the children may be significant in giving meaning to life (Mueser & Fox, 2002; Trulsson & Hedin, 2004). It could be argued that the service system should constantly have the family aspect as a basis of their work. Supporting a family perspective alongside individual support is an important, but an unsystematically used resource (Drake et al., 2001; Minkoff, 2006; Trulsson & Hedin, 2004).

### The staff perspective

Several events occurred before the rehabilitation process started. Engagement occurred after several years. Where did it start? In this study, it was not possible to differentiate the role of the different service providers. Processing the life events started during psychiatric hospitalization. The connection grew out of a personal and trustful connection to a primary nurse. The social workers’ role was strong in the form of a child being taken into care, and in challenging the self-determination of the client. The data reveal that the process of recovery is extremely complex, and that the carers need a sensitive and appreciative approach (Howell et al., 1998). The relationship with keyworkers can redirect the process of change (Trulsson & Hedin, 2004). For staff, it is crucial to listen more intently to the individual clients, and staff should find the means to reach all clients. More research is needed to comprehend the individual barriers in help-seeking.

The emotions arising during contacts with services can perhaps impede collaboration with staff. The drug abuse in the family (Walton-Moss & Becker, 2000) may raise fear, as well as the background of abuse and experienced violence (Rosenbaum, 1979). The negative and ambivalent emotions such as guilt, resentment, and anxiety may be too much to cope with, and thus the solution of the client may be to drop out from caring experiences. It is unclear how much cultural Finnish perceptions of motherhood had an impact on the decisions made, our participant claimed she wanted to be “the perfect mum,” but did not want anybody to interfere, and wanted to be let alone for a long time.

## Conclusion

This article explored and described the lived experiences, intentions, and motives in help-seeking from the perspective of a mother with a dual diagnosis. The essential meaning structure is an inner conflict within the client, including a realization that change starts from within. The essential meaning structure combines the other meaning structures: disbelief of receiving help and admitting the need for help, keeping up the perfect façade and the risk of total collapse, being given and making own choices regarding care and being forced to use services, inner emptiness and search for contentment in life.

It is possible that clients in the help-seeking process do not always recognize they have any problems. If the client experiences inner powerlessness as emptiness and resistance to being helped, it is probably not possible to create relationships with care providers. Clients may have several ambiguous inner processes, which prevent them from accepting the need for care. The data also contained situations where the client found solutions outside the services.

Theoretically and empirically a long-term approach is crucial, since the inner transformative processes take time. An essential question is whether the fragmented service delivery system uses sufficient communication and networking to enhance the care of the dually diagnosed mothers? The data showed that substance abuse assessment was not a common feature. The verbal level, giving information, does not seem enough, if the idea is to identify those in need as early as possible. The services should be developed into a more inclusive policy, with collaborative skills across different sectors.

Possible tools include training professionals in using standard screening tools, or using continuous and family-centred care models. A supportive family health care focus, including a thorough mental health assessment, with a focus on the client history and future goals would help this client group. Personal relationships with keyworkers may enhance the changes. Most importantly, the services can contribute new experiences to the personal level of understanding and decision-making, if they consider the experiential level of their clients.
